# Supplementary material for: Subduction initiation triggered the Caribbean large igneous province
Source: Nat Commun. 2023 Feb 11;14:786. doi: 10.1038/s41467-023-36419-x (PMC9922256; doi:10.1038/s41467-023-36419-x)
Supplement: Supplementary file 1 — Supplementary Information [file 41467_2023_36419_MOESM1_ESM.pdf]

Subduction initiation triggered the Caribbean Large Igneous Province

Nicolas Riel[1,2*], João Duarte[3], Jaime Almeida[3], Boris J.P. Kaus[1,2], Filipe Rosas[3], Yamirka Rojas-Agramonte[1,4] and Anton Popov[1]

[1*]Institute of Geosciences, Johannes Gutenberg-University, Mainz, Germany.
[2*]Terrestrial Magmatic Systems (TeMaS) research center, Johannes Gutenberg-University, Mainz, Germany.
[3*]Instituto Dom Luiz, University of Lisbon, Lisbon, Portugal.
[4*]Institute of Geosciences, University of Kiel, Kiel, Germany.

*Corresponding author(s). E-mail(s): nriel@uni-mainz.de;

1 Supplementary Information

1.1 Modelling results

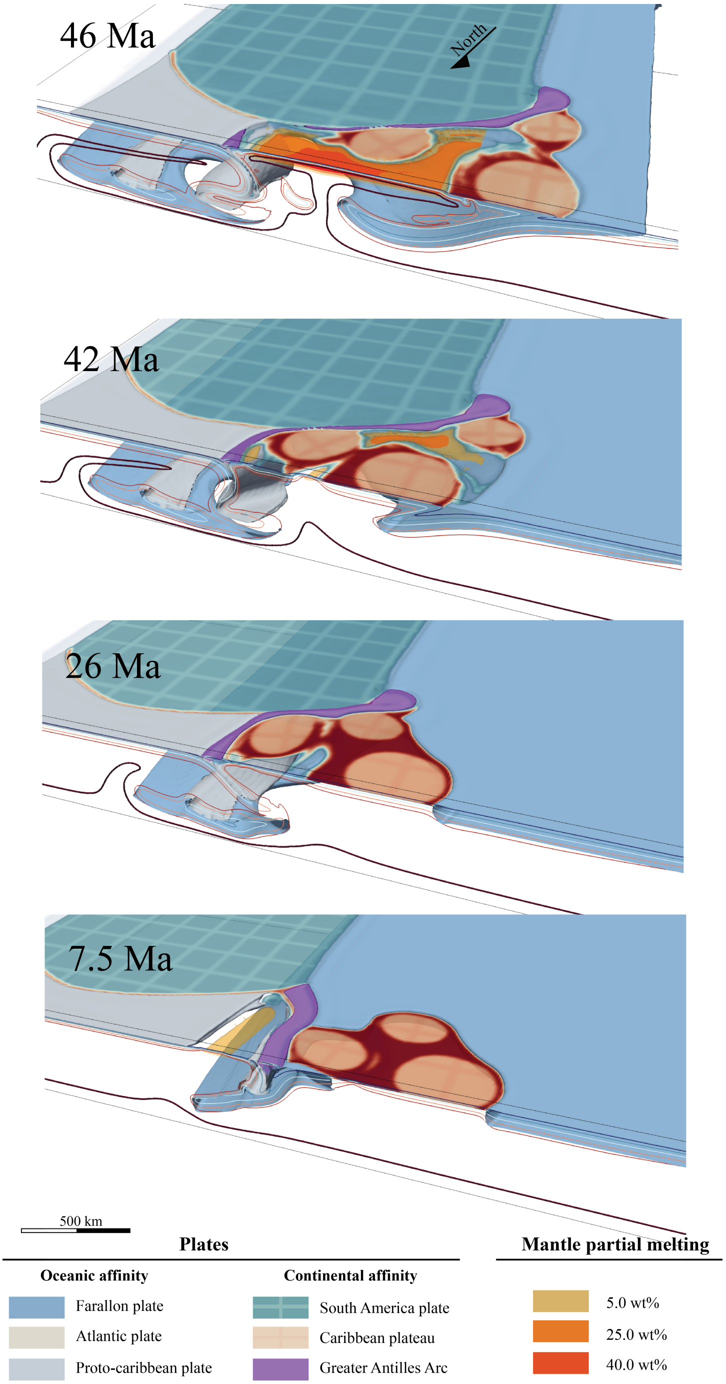

Supplementary Fig. 1 Model SP.1

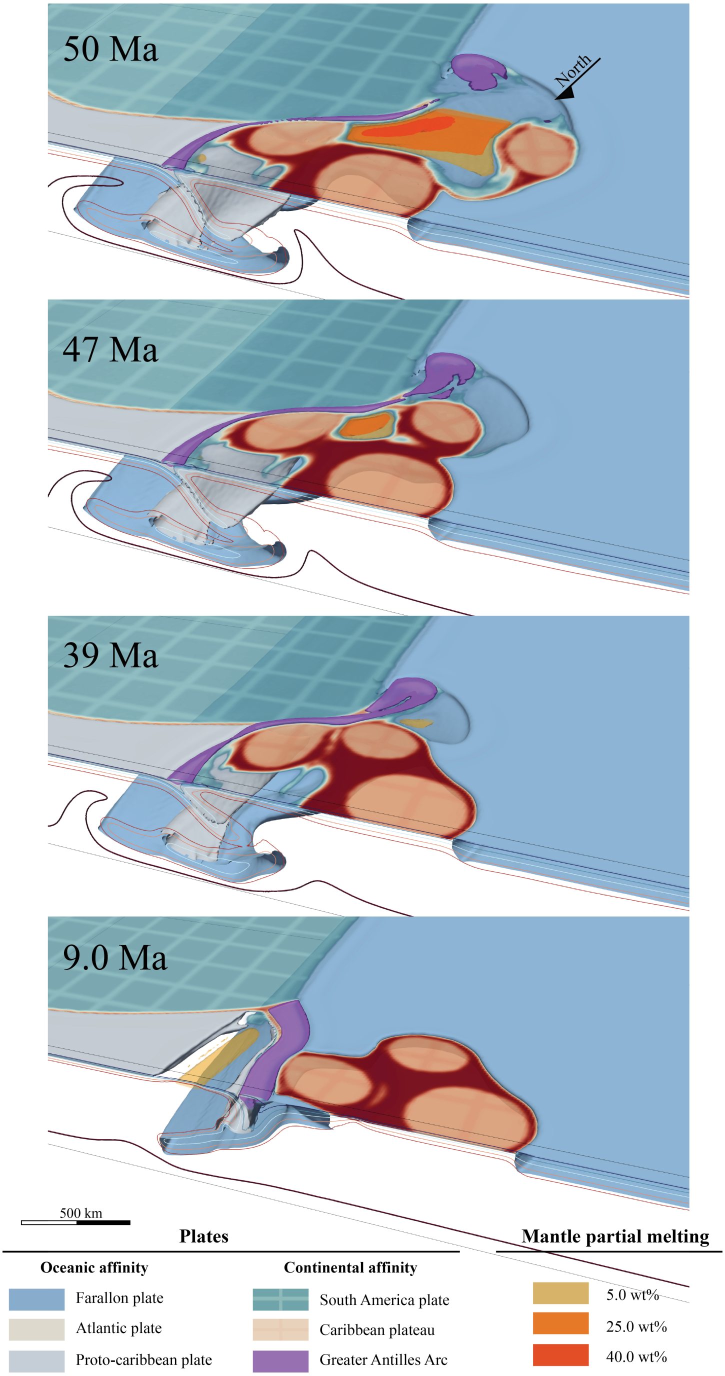

Supplementary Fig. 2 Model SP.2

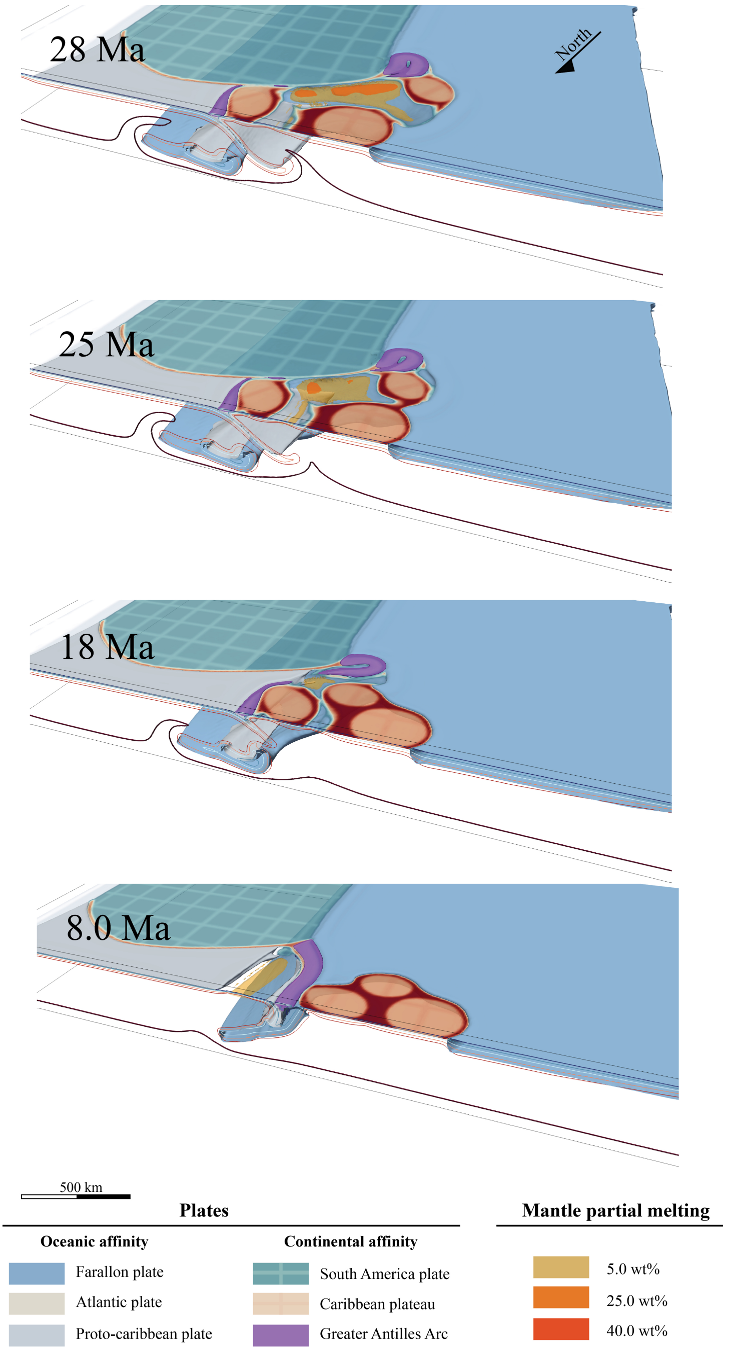

Supplementary Fig. 3 Model SP.3

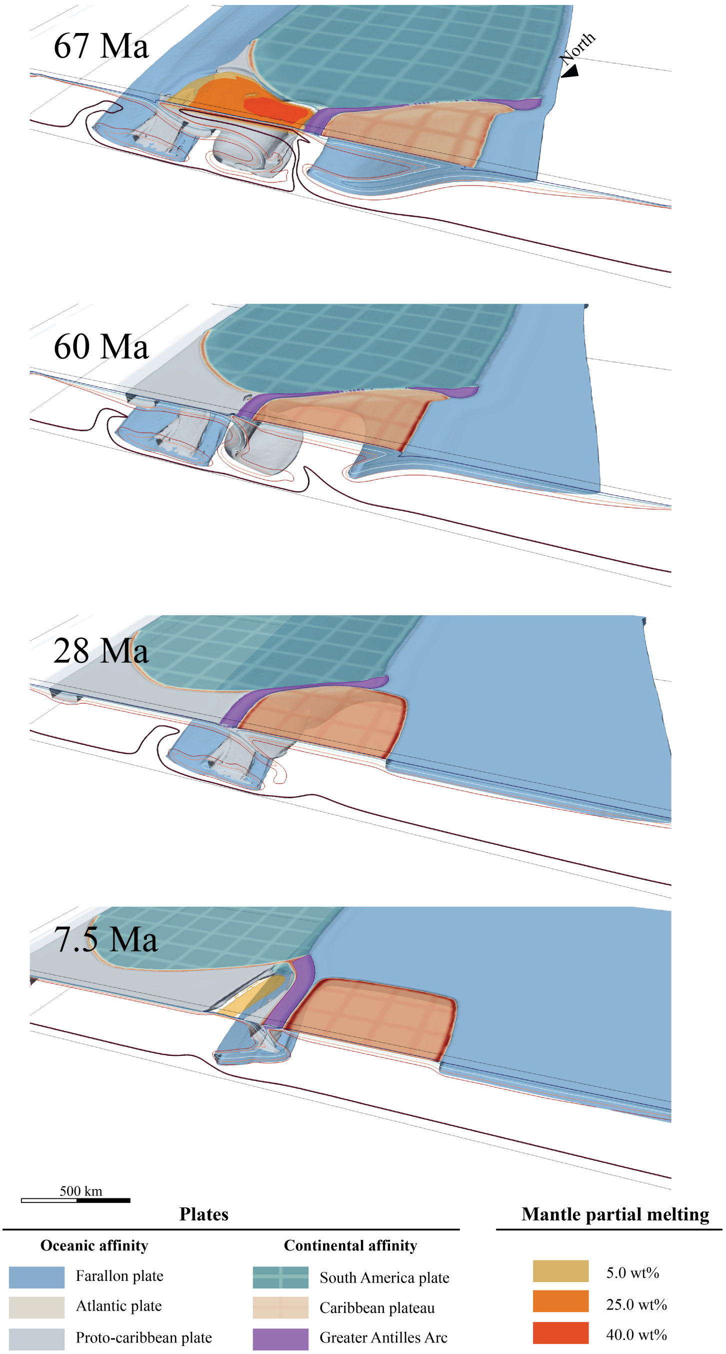

Supplementary Fig. 4 Model LP.1

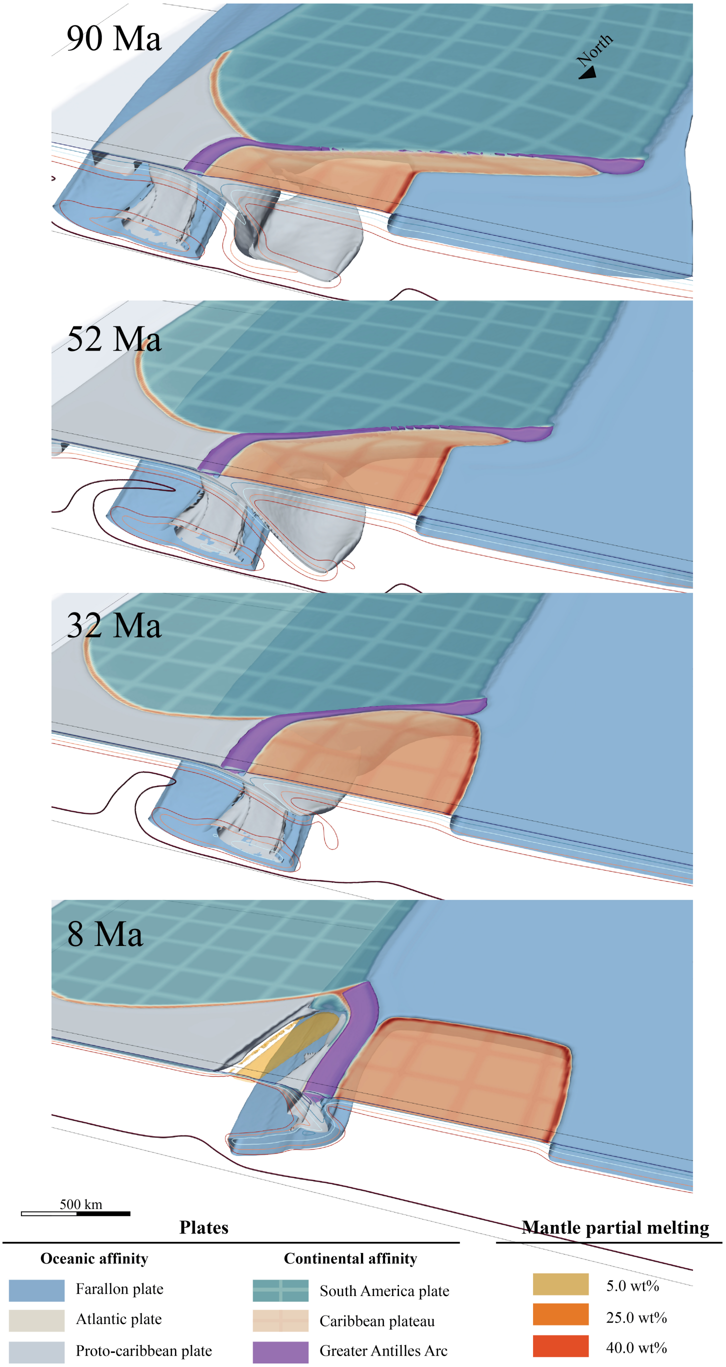

Supplementary Fig. 5 Model LP.2

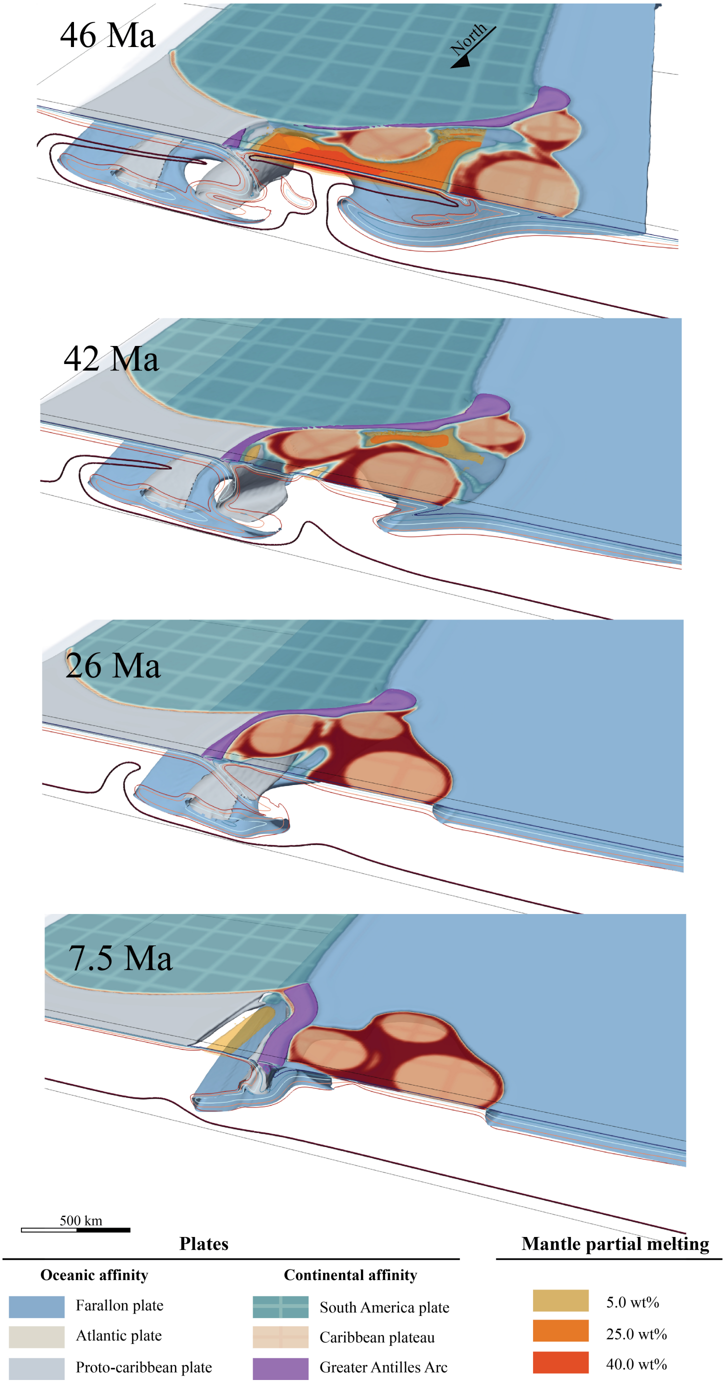

Supplementary Fig. 6 Model CP.1

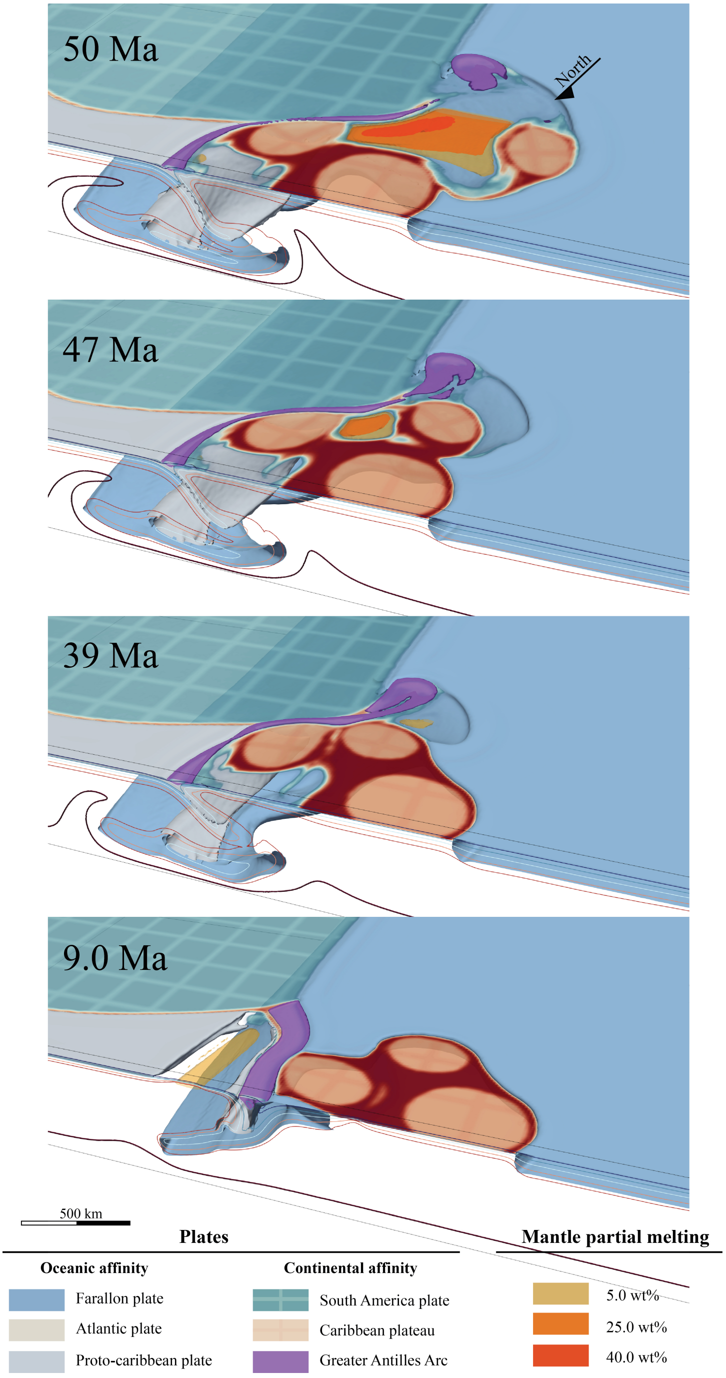

Supplementary Fig. 7 Model CP.2

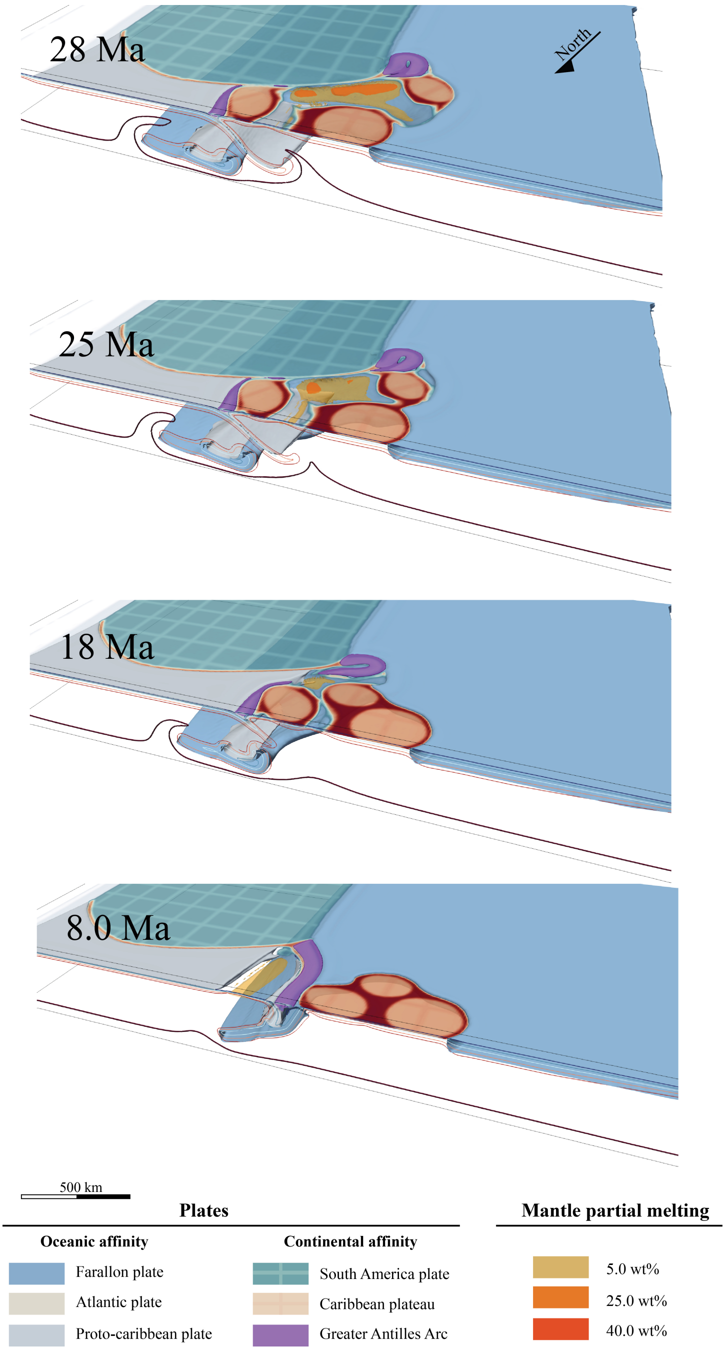

Supplementary Fig. 8 Model CP.3

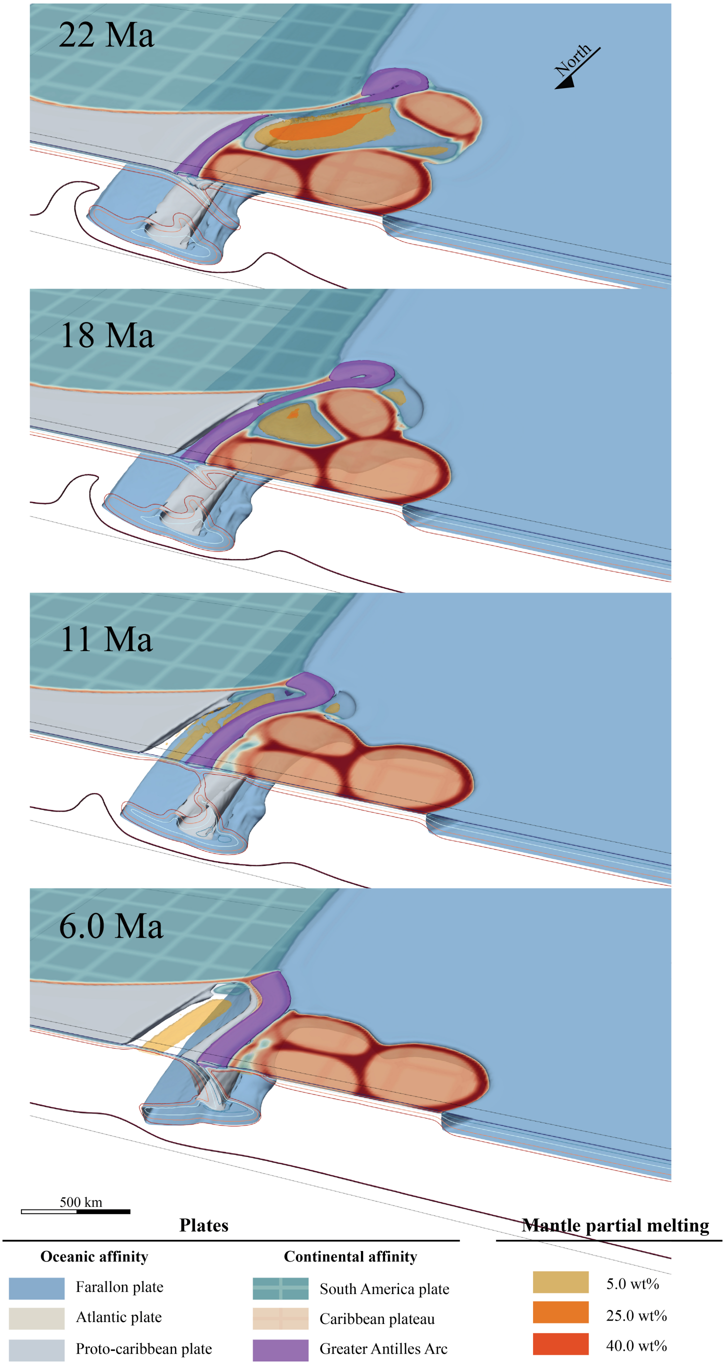

Supplementary Fig. 9 Model CP.4

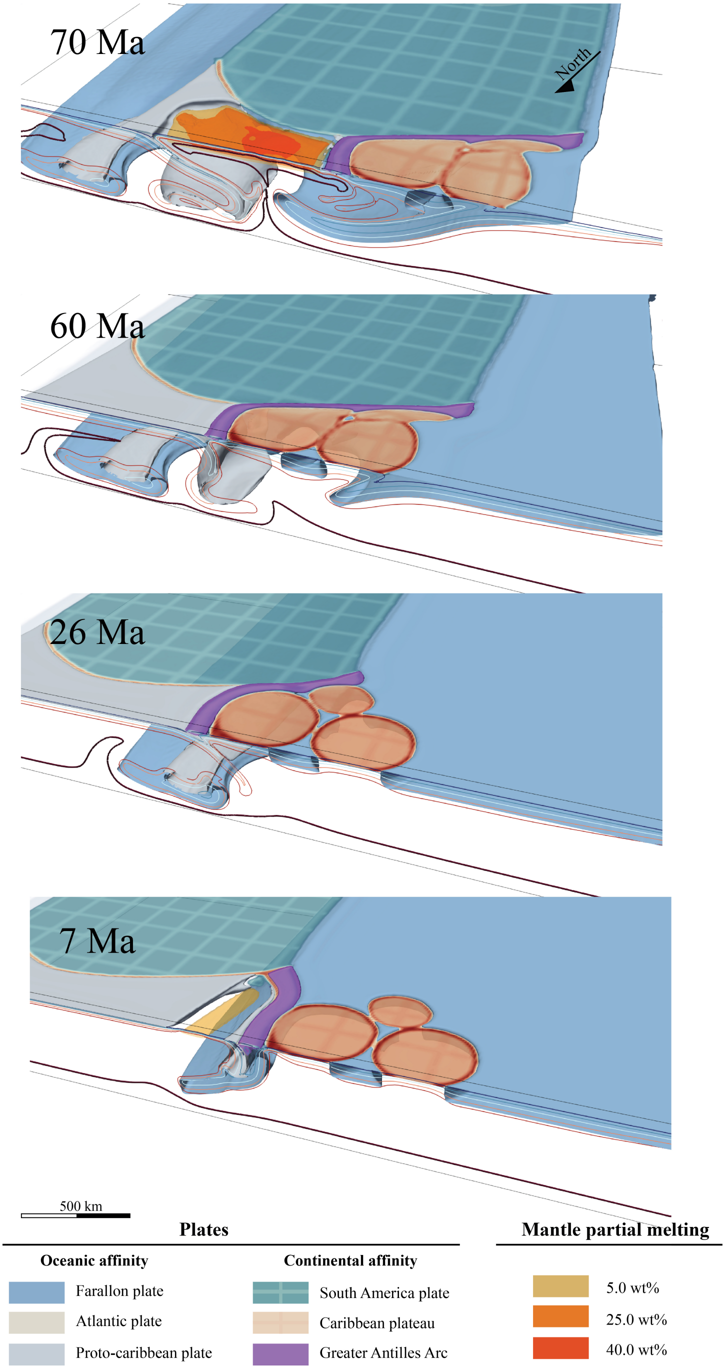

Supplementary Fig. 10 Model CP.5

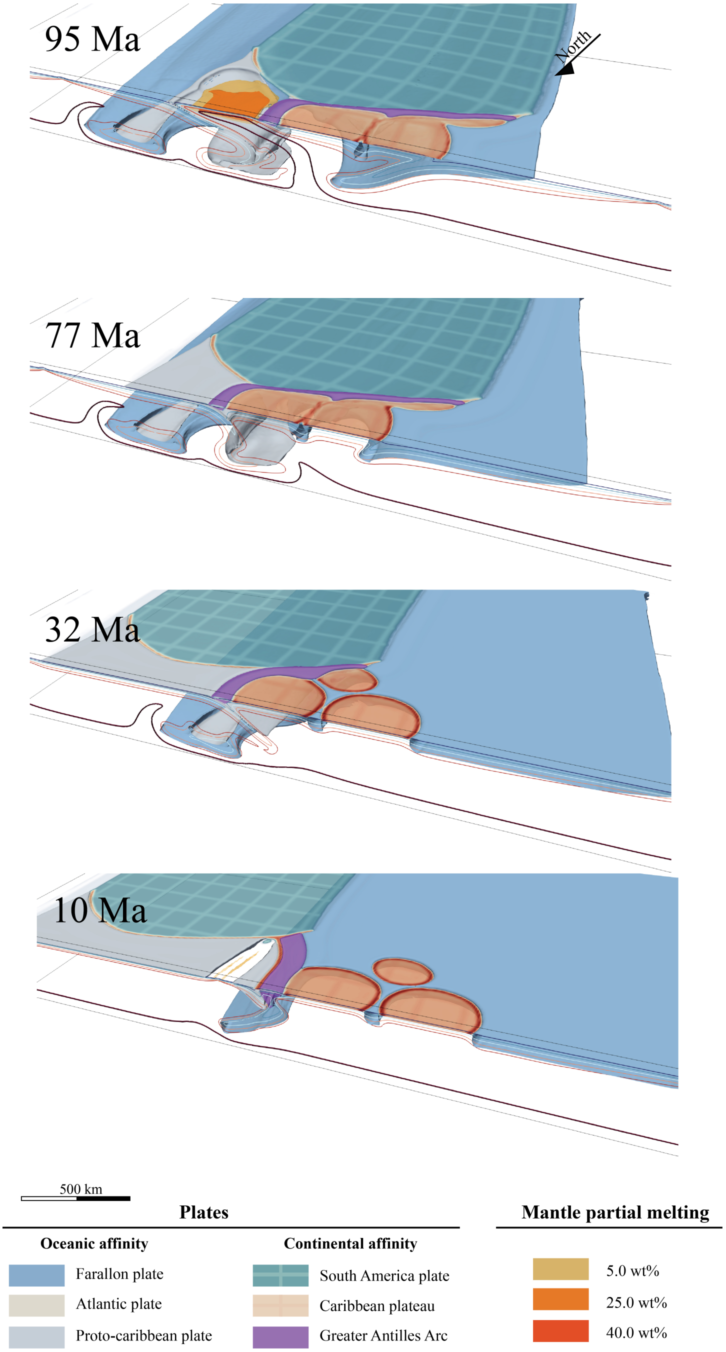

Supplementary Fig. 11 Model CP.6

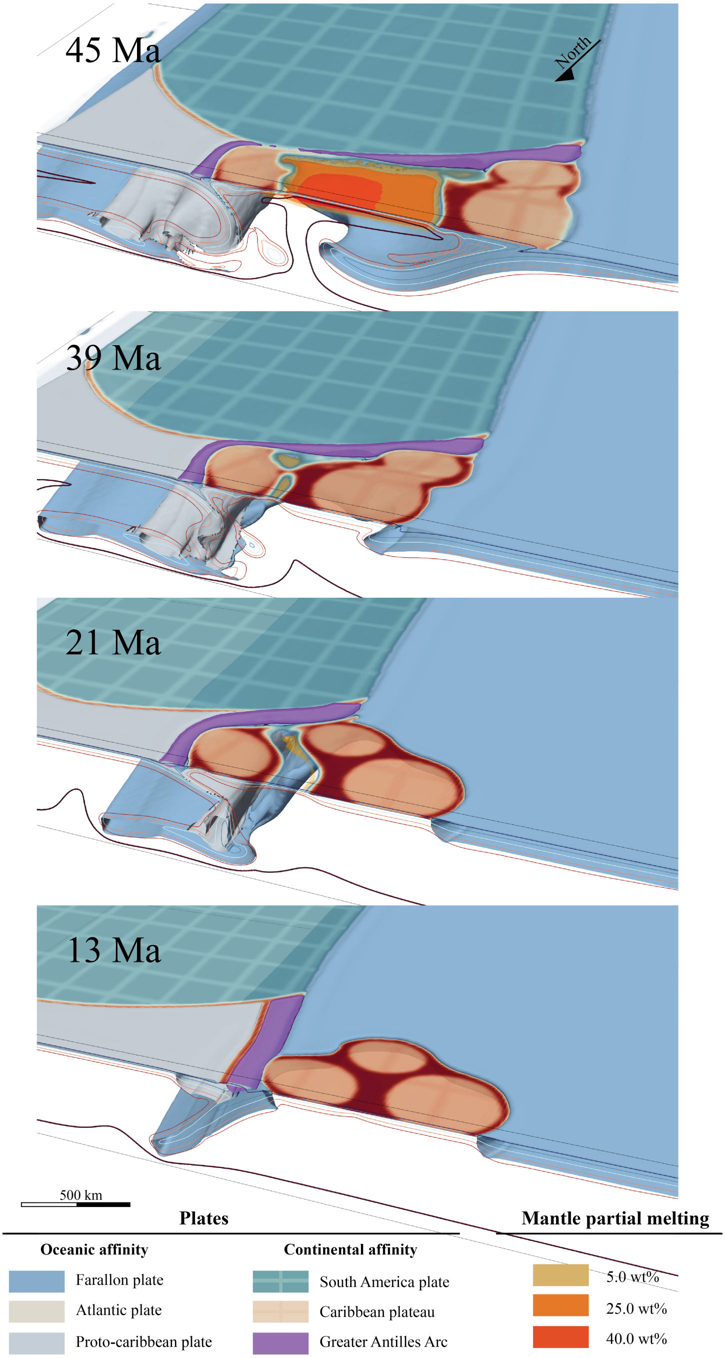

Supplementary Fig. 12 Model CP.7

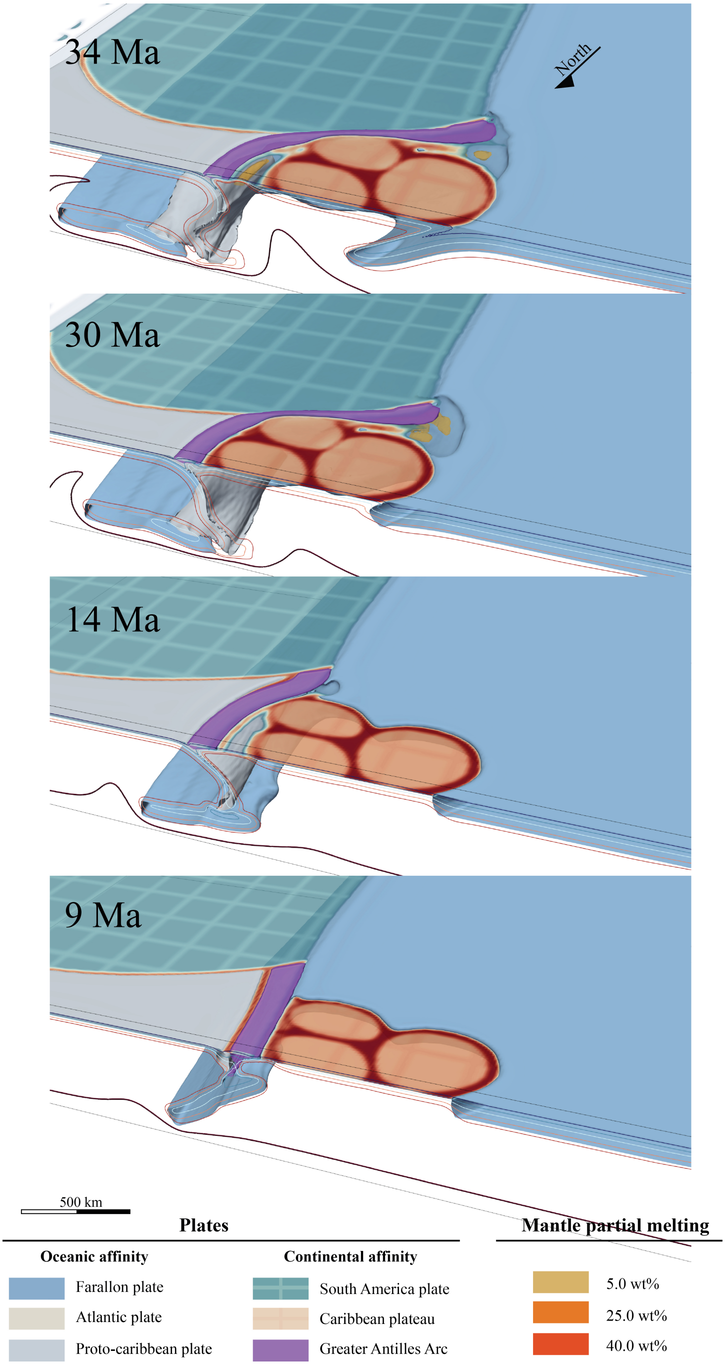

Supplementary Fig. 13 Model CP.8

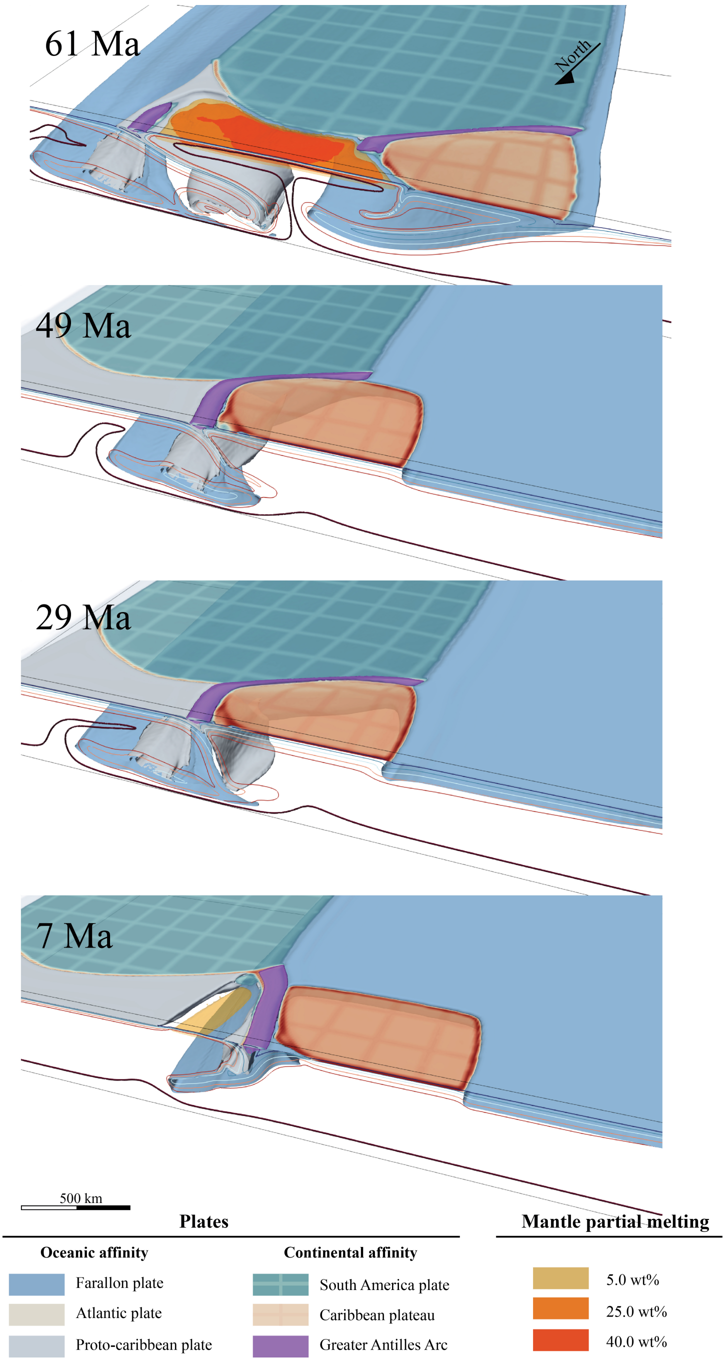

Supplementary Fig. 14 Model MP.1

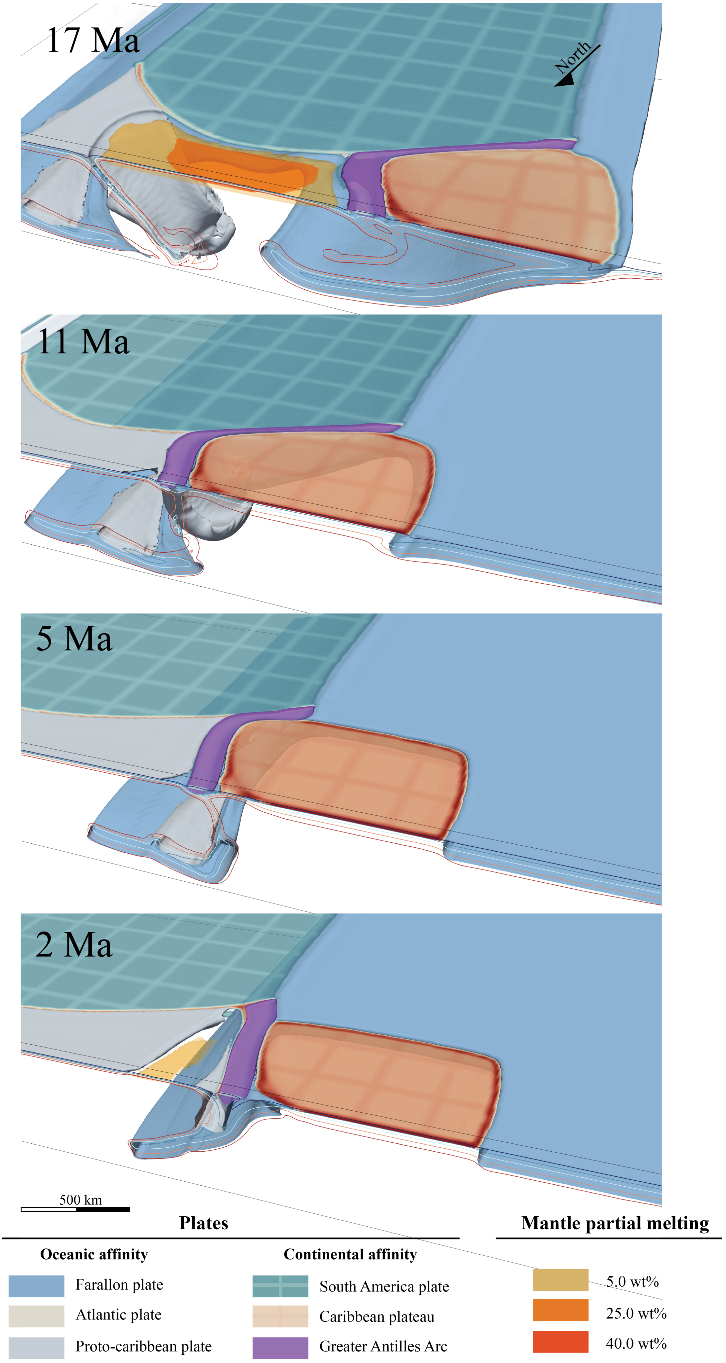

Supplementary Fig. 15 Model MP.1a

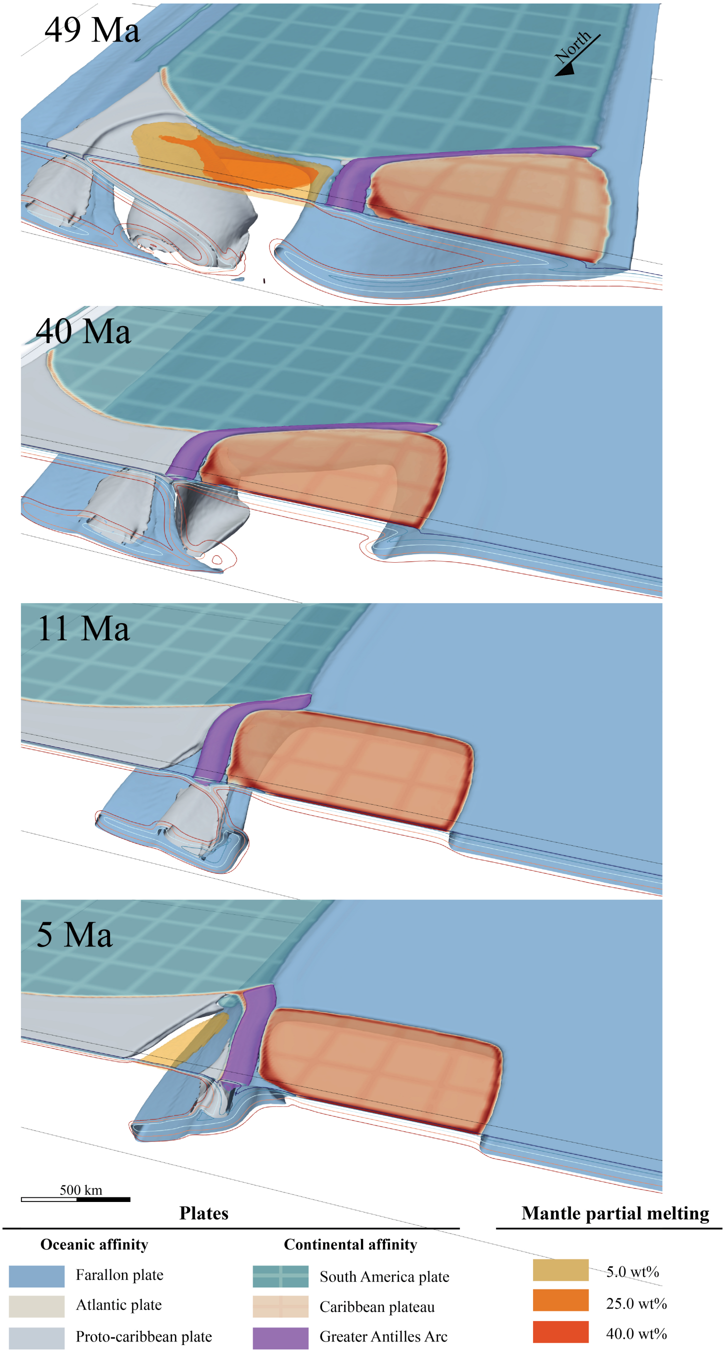

Supplementary Fig. 16 Model MP.1b

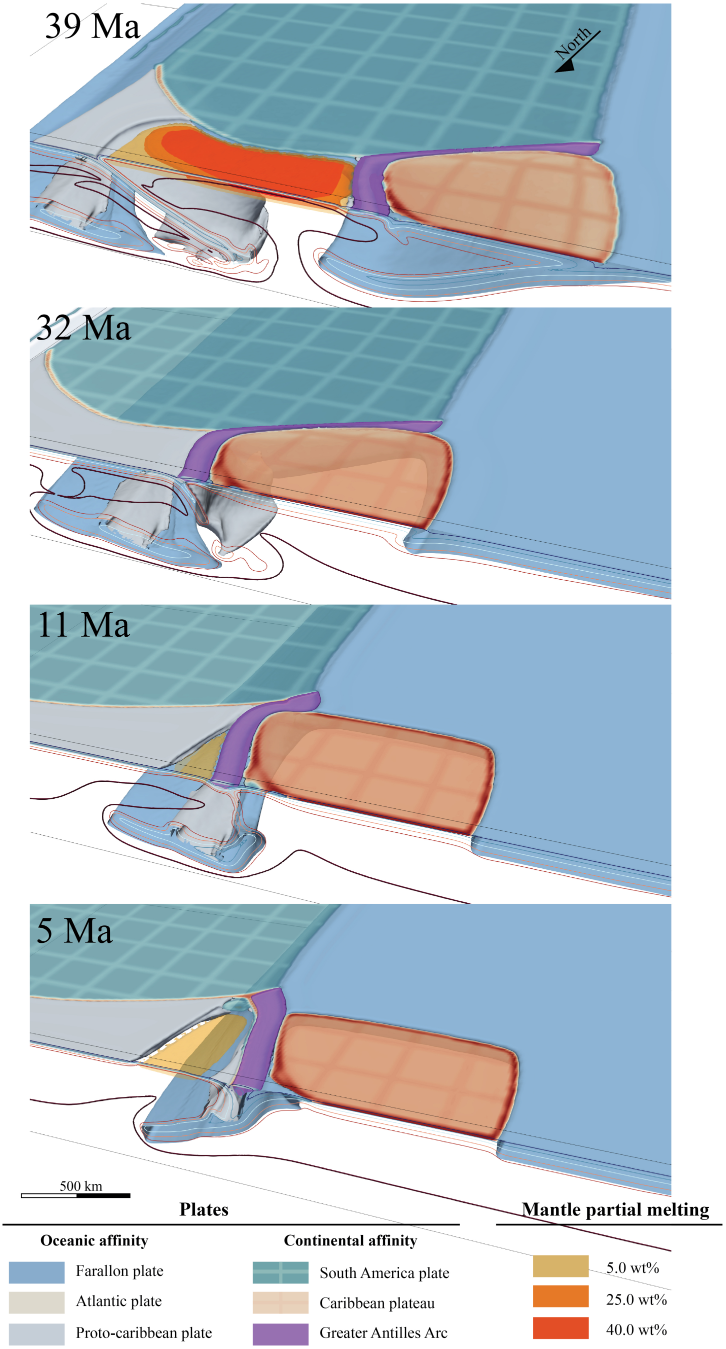

Supplementary Fig. 17 Model MP.1c

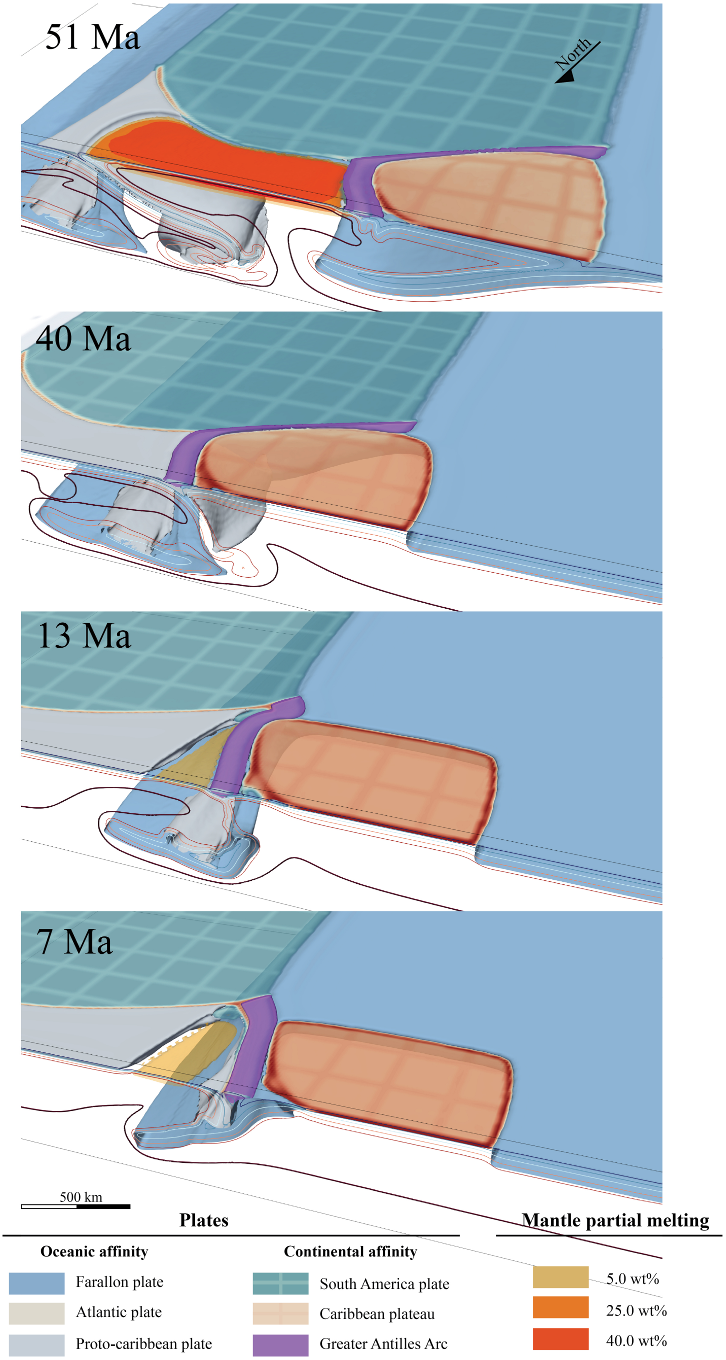

Supplementary Fig. 18 Model MP.1d

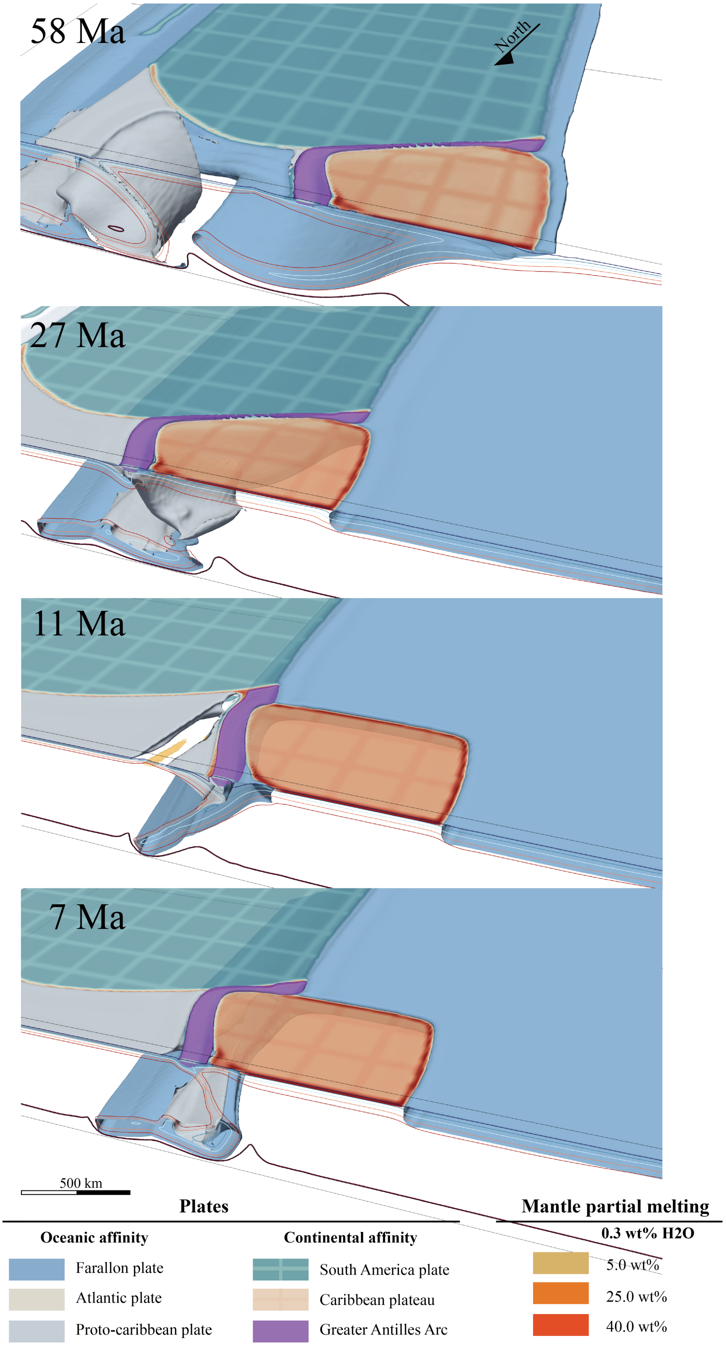

Supplementary Fig. 19 Model MP.1AH

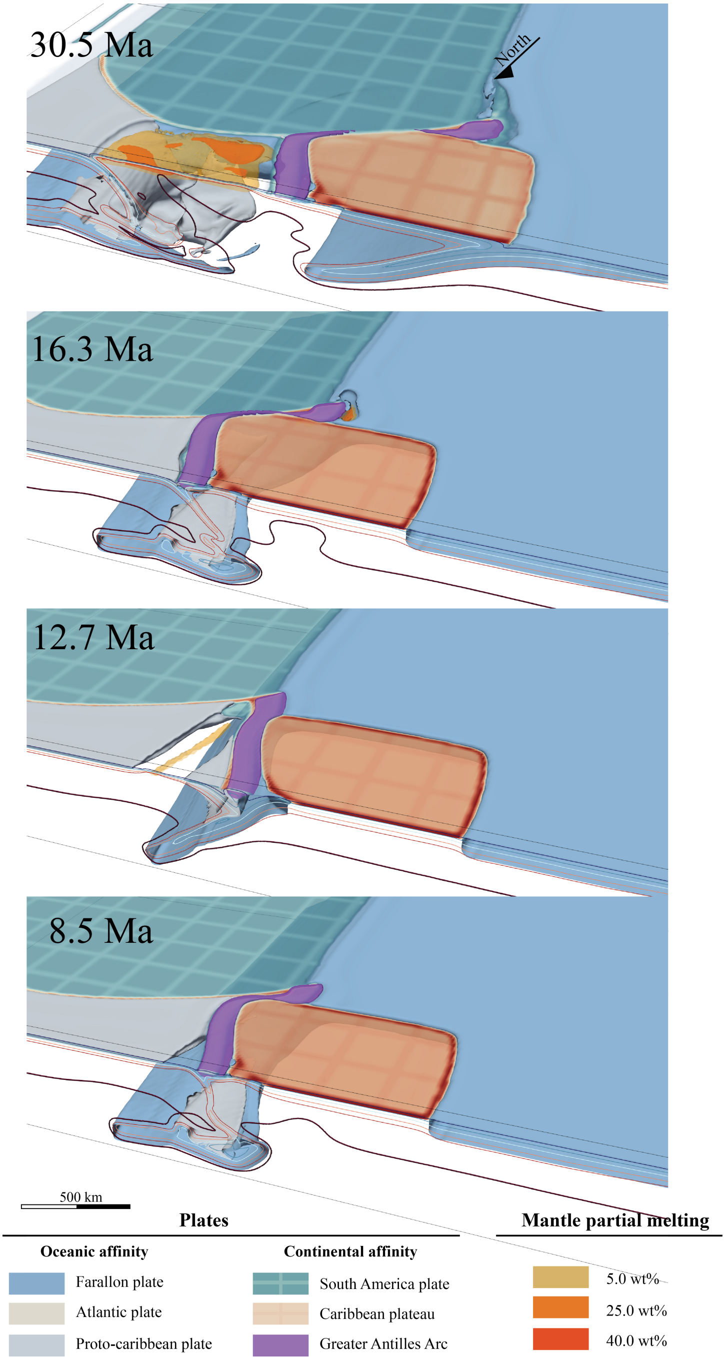

Supplementary Fig. 20 Model MP.AH.HA.1

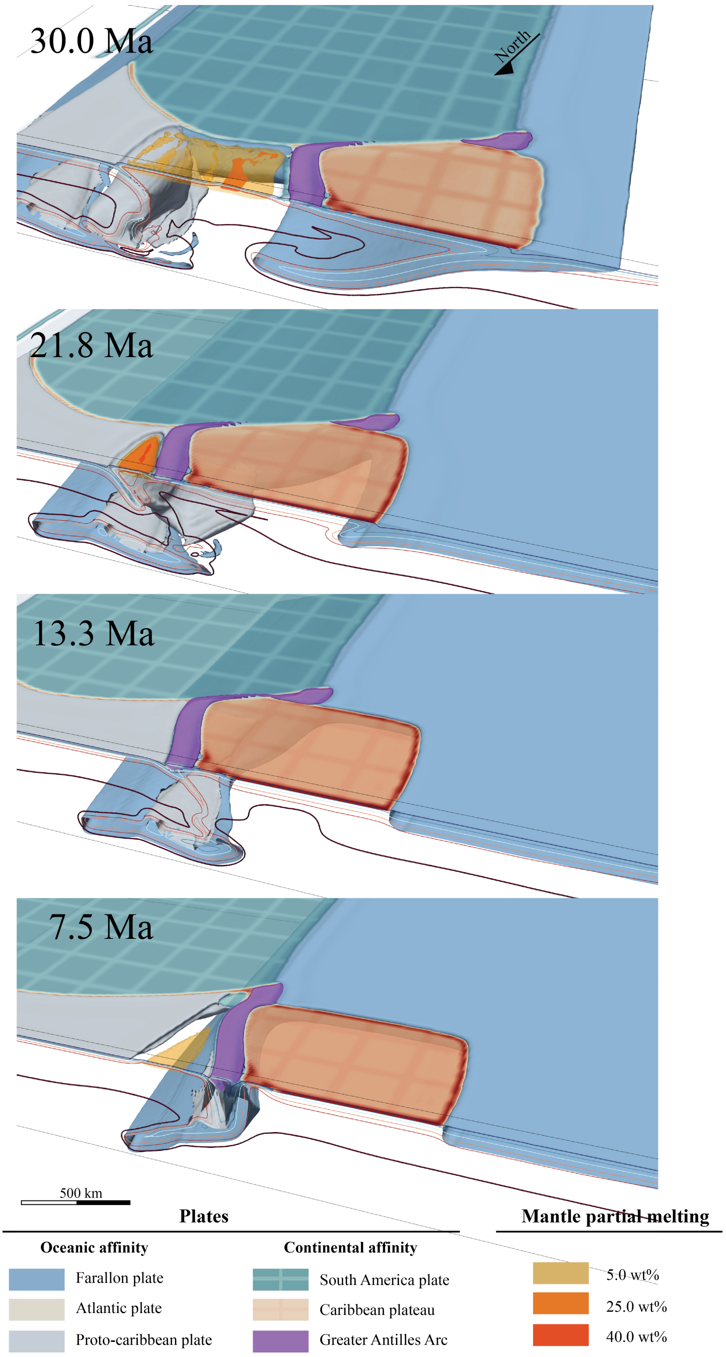

Supplementary Fig. 21 Model MP.AH.HA.2

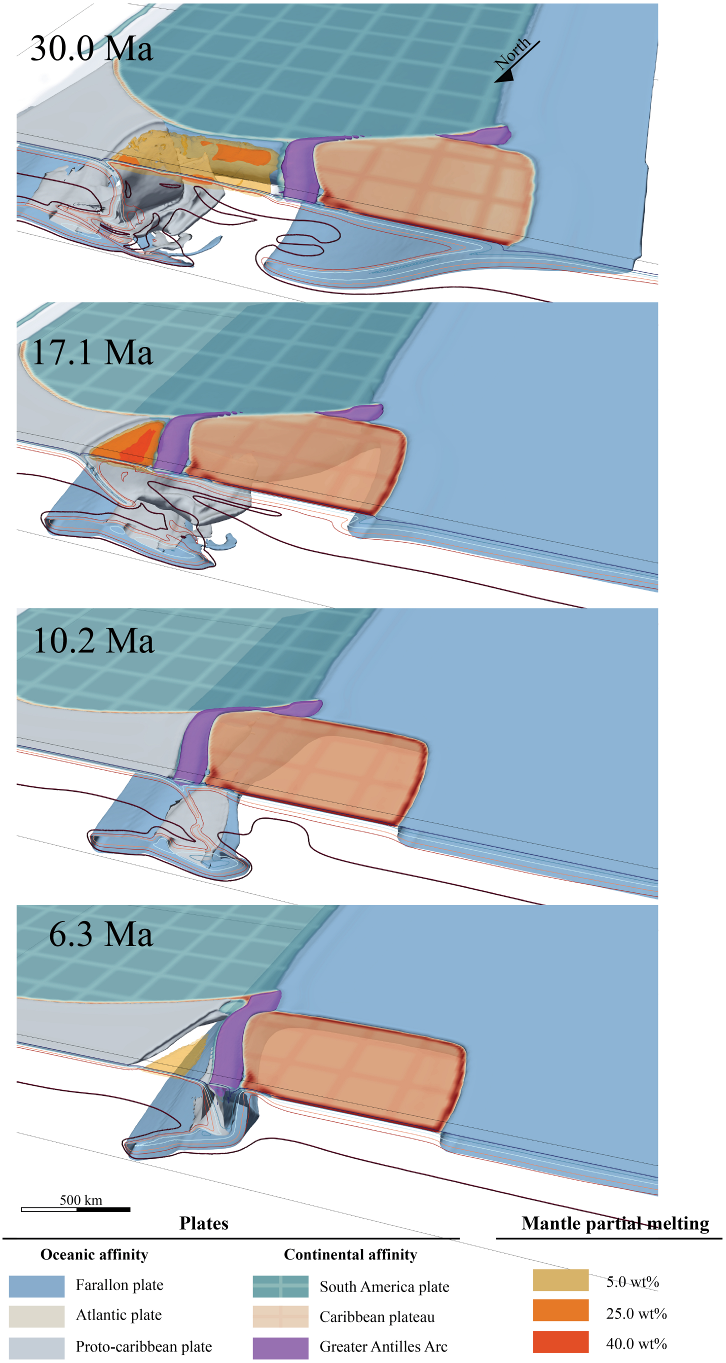

Supplementary Fig. 22 Model MP.AH.HA.3

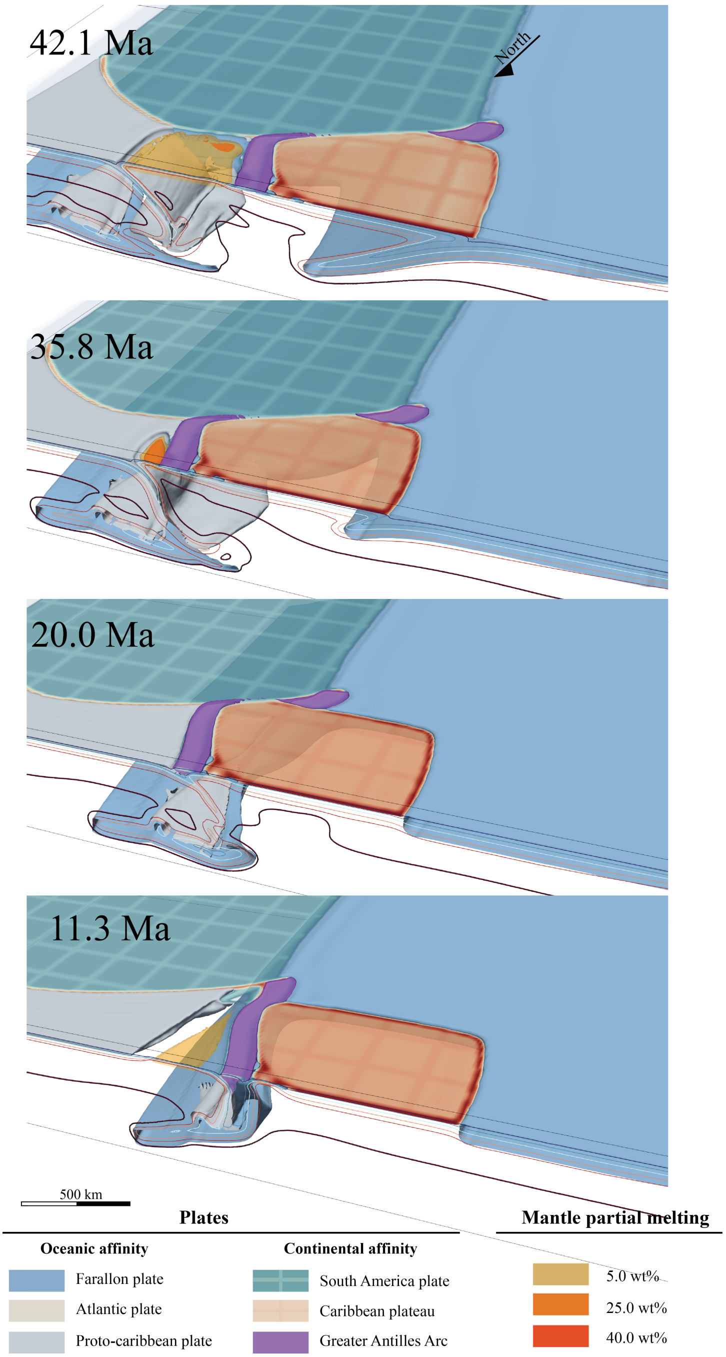

Supplementary Fig. 23 Model MP.AH.HA.4

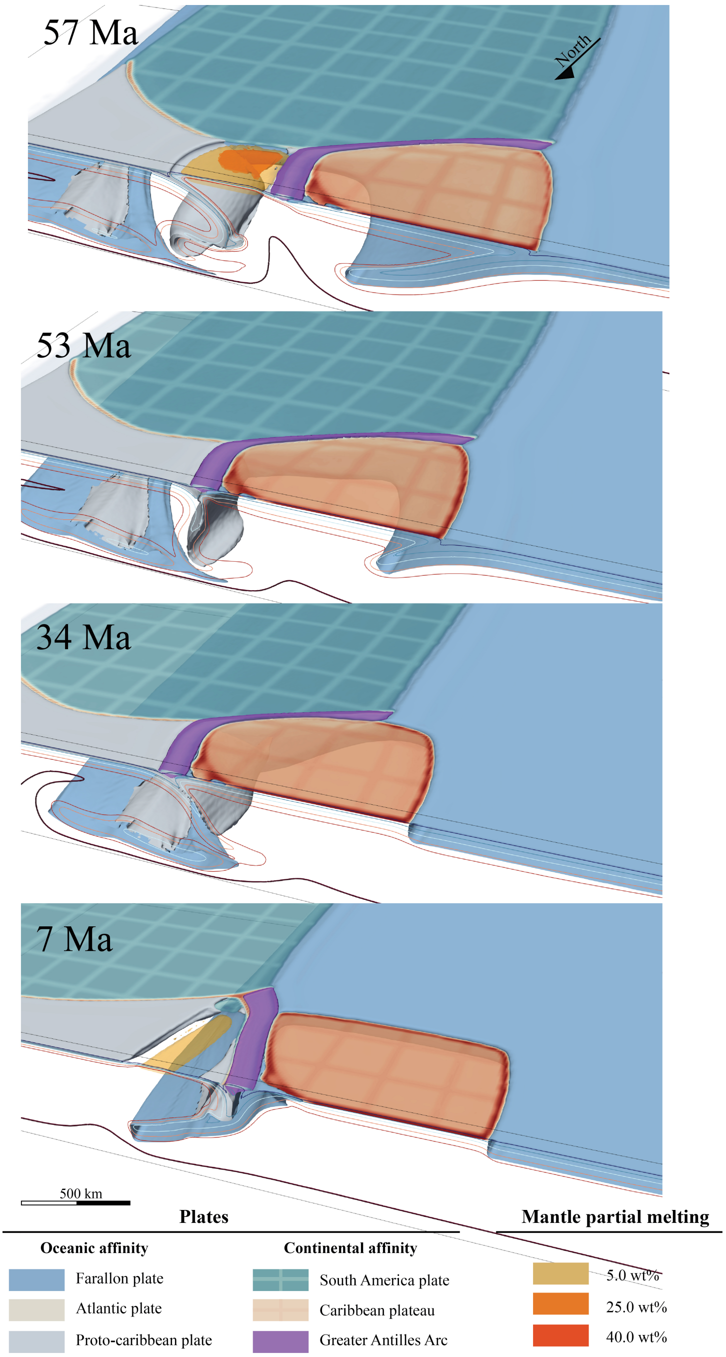

Supplementary Fig. 24 Model MP.2

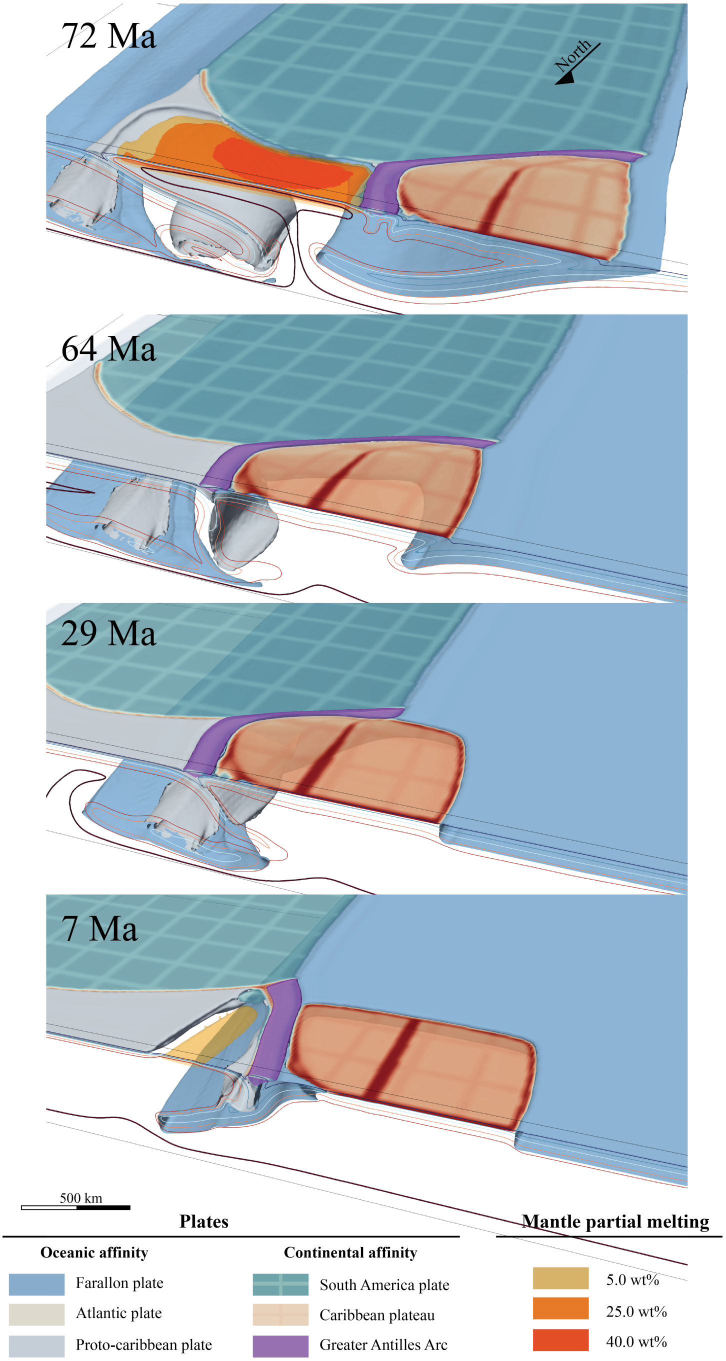

Supplementary Fig. 25 Model MP.3

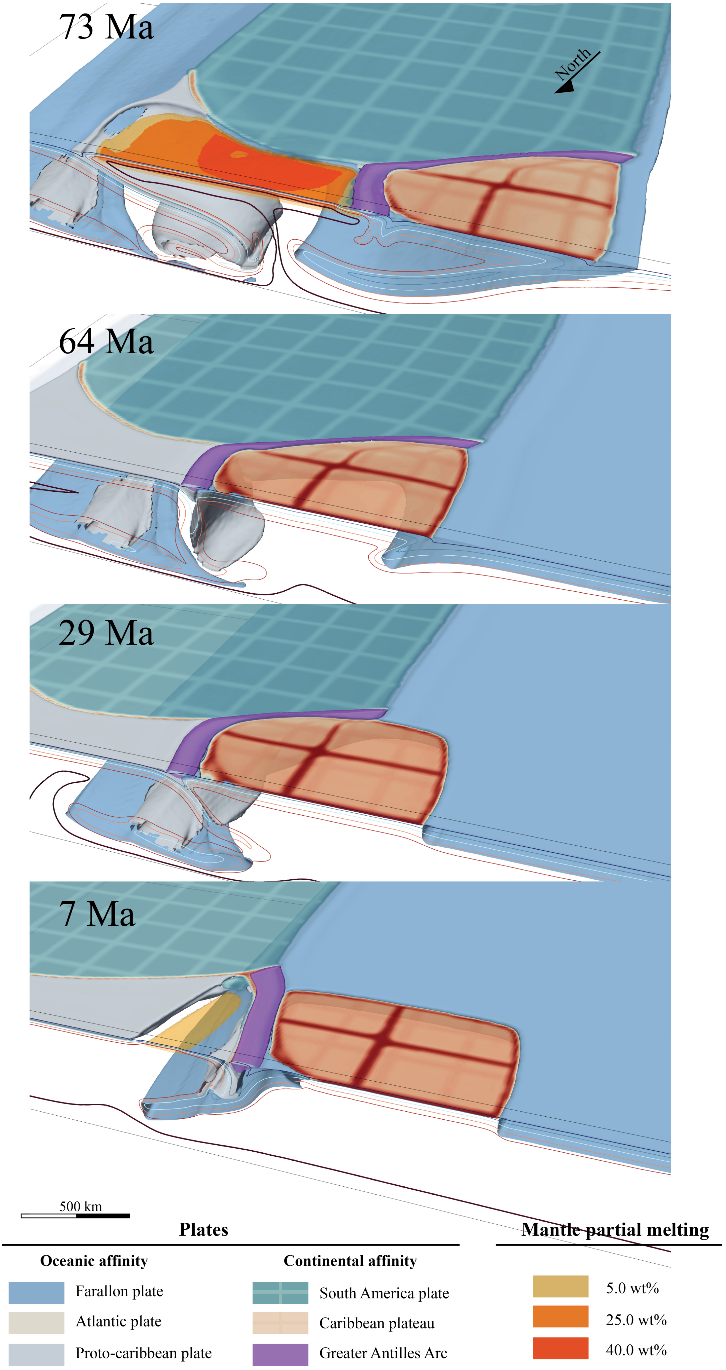

Supplementary Fig. 26 Model MP.4

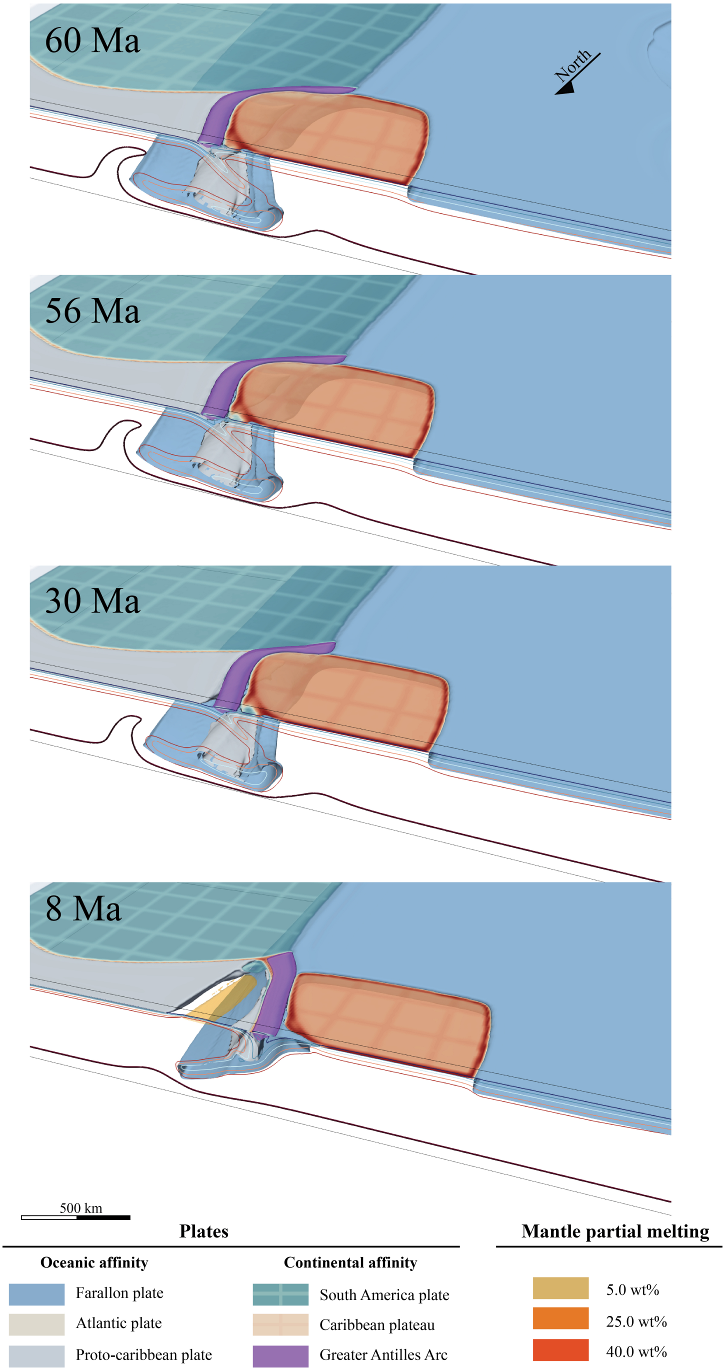

Supplementary Fig. 27 Model MP.5

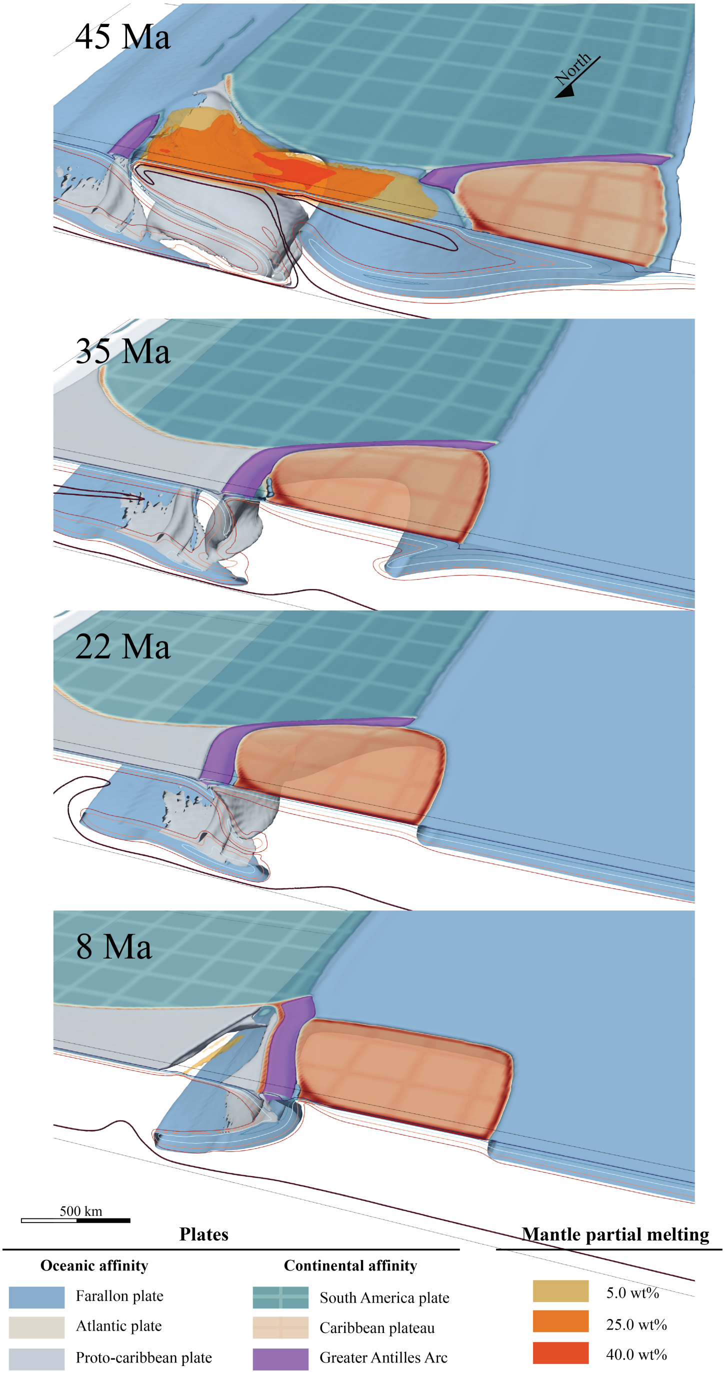

Supplementary Fig. 28 Model M2P.1

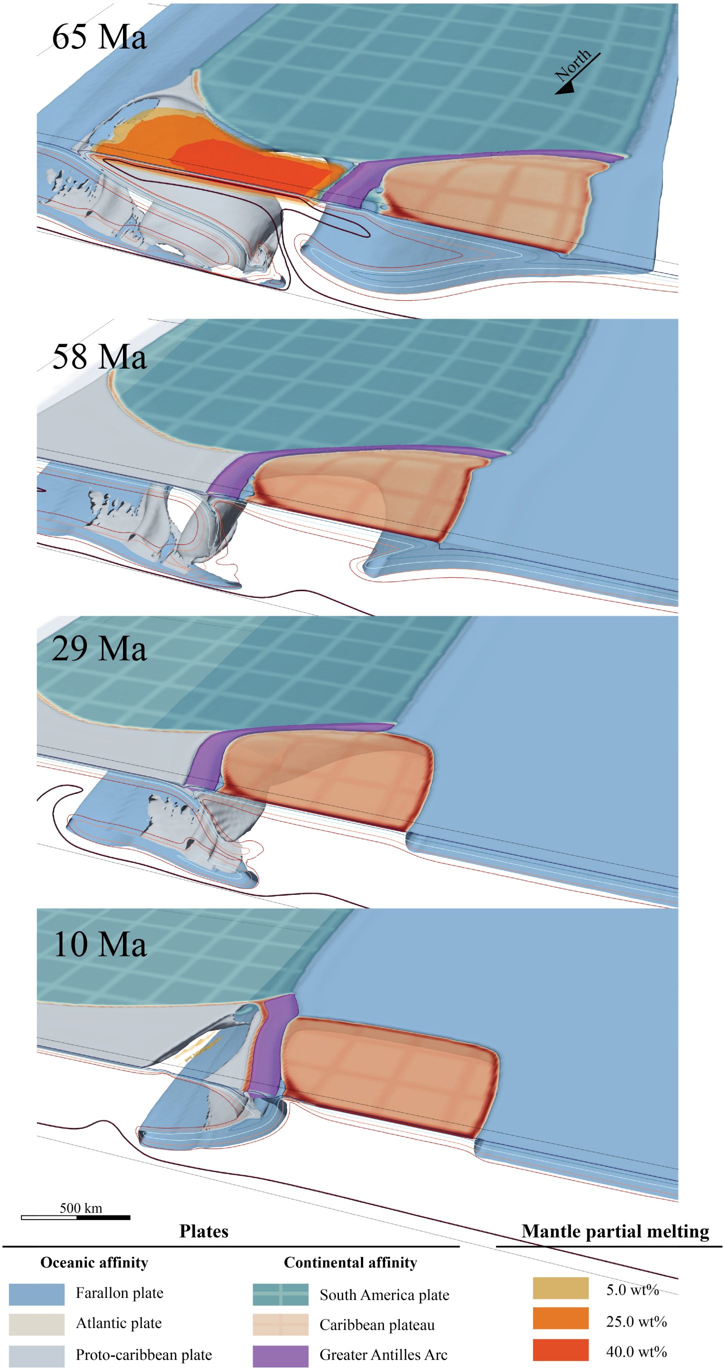

Supplementary Fig. 29 Model M2P.2

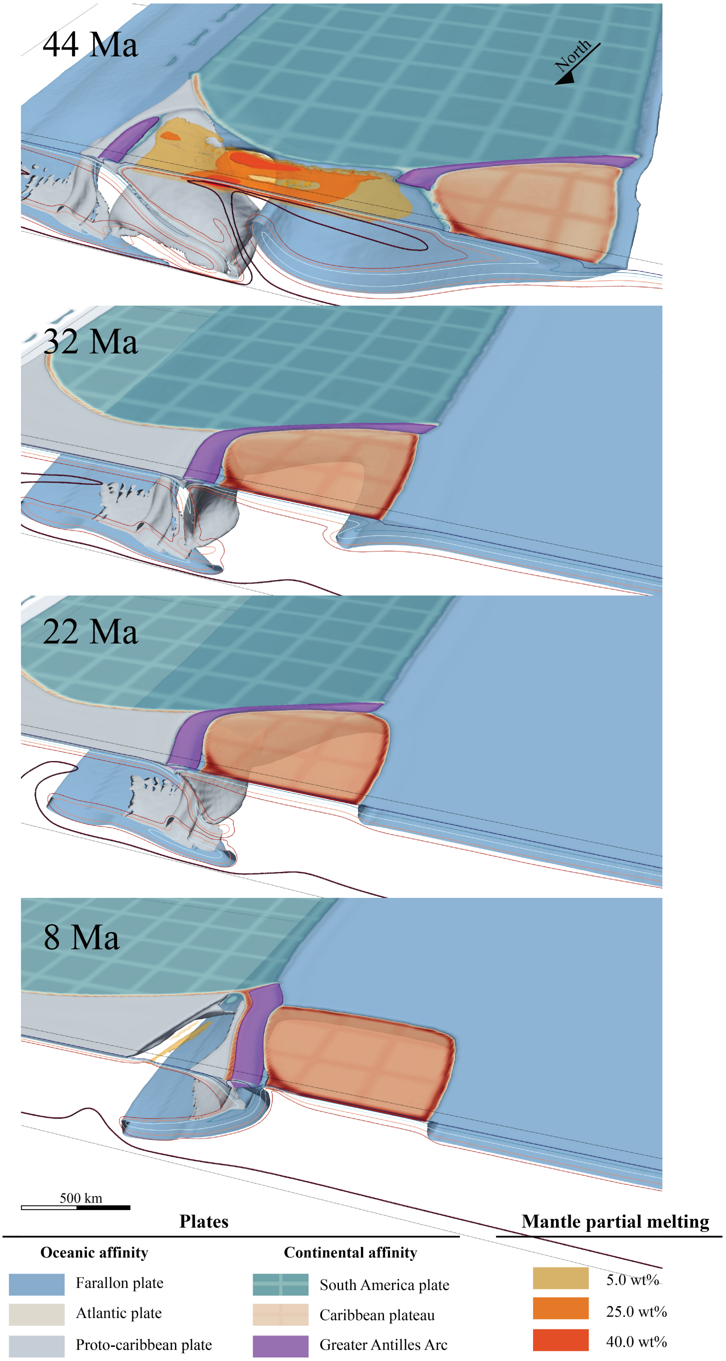

Supplementary Fig. 30 Model M2P.3

1.2 Role of the initial mantle temperature

Simulations MP.1a and MP.1b show the role of a lower initial temperature gradient for the upper mantle of 0.2°C.km^{-1} reaching a maximum temperature of 1460 °C at the bottom wall of the model (see Fig. 1C). We find that both simulations result in a significant decrease of the predicted volume of partial melting with respect to the reference model (with an upper mantle temperature gradient of 0.3°C.km^{-1}). While model MP.1a (Fig. 15) yields a small volume of excess magma of 1.5×10^4 km^3, for model MP.1b (Fig. 16), no excess volume of magma is predicted (Fig. 31). This difference is explained by having a significantly lower activation volume for model MP.1a (11×10^{-6} m^3.mol^{-1}) compared to MP.1b (12.25×10^{-6} m^3.mol^{-1}). The effect of activation volume is also well illustrated by the change in simulation time: 17 Ma for model MP.1a (Fig. 15) and a more realistic simulation time of 49 Ma for model MP.1b (Fig. 16).

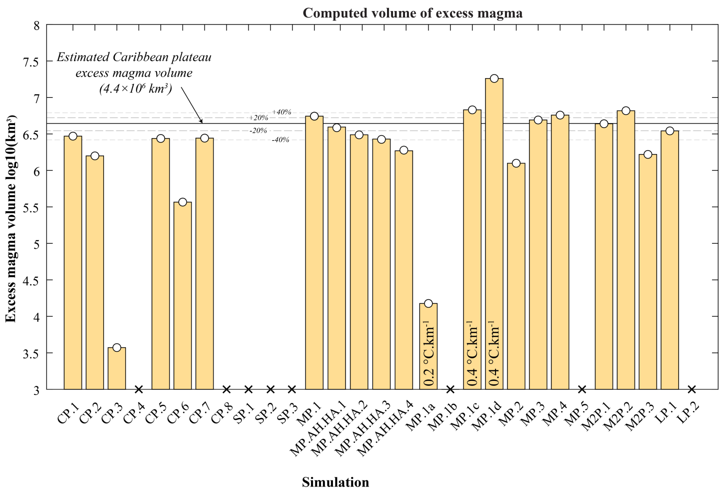

Supplementary Fig. 31 Calculated excess magma volume

Simulations MP.1c and MP.1d (Figs 17 and 18) explore the role of higher initial temperature gradient for the upper mantle of 0.4°C.km^{-1} reaching a maximum temperature of 1600 °C at the bottom wall of the model (see Fig. 1C). Higher mantle temperature scaled to comparable geodynamic time by using higher activation volume led to higher partial melting during subduction-induced plume formation (Figs 17 and 18). The resulting calculated volume of excess magma yield value ranging from 6.7×10^6 to 1.8×10^7 km^3 (Fig. 31), 1.5 to 4 times higher than the estimated value for the Caribbean plateau of 4.4×10^6 km^3[1].

1.3 Boussinesq versus extend Boussinesq approximation

In simulations MP.AH.HA.1 to 5 (figures 20 to 23) we explore the role of using the extended Boussinesq approximation instead of using the Boussinesq approximation (such as for the reference models). We find that the simulations using the extended Boussinesq approximation yield very similar geodynamic evolution than simulations using only the Boussinesq approximation. This includes collision of the old plateau with the proto-Caribbean margin triggering subduction polarity reversal, transfer of the GAA and the old Caribbean along the northern margin of the south American plate, renewal of the Farallon plate subduction on the back on the accreted plateau and subduction-driven plume formation between the retreating proto-caribbean subduction plate and the front of the old caribbean plateau (figures 20 to 23). Moreover, we find that the computed volume of excess magma are of the same order of magnitude to estimated value of 4.4×10^6 km^3[1] (Fig. 31), thus supporting the robustness of our approach.

1.4 Greater Antilles Arc transfer

One interesting feature presented in the reference model (MP.1, Fig. 14) is the partial transfer of the Greater Antilles Arc from the eastern edge of the Caribbean plateau onto the rolling back proto-Caribbean plate during the early stage of the plume formation (Fig. 14). This feature is observed in simulations SP.1 (Fig. 1), SP.2 (Fig. 2), SP.3 (Fig. 3), CP.1 (Fig. 6), CP.2 (Fig. 7), CP.3 (Fig. 8), CP.7 (Fig. 12), CP.8 (Fig. 13), MP.1 (Fig. 14), M2P.1 (Fig. 28) and M2P.3 (Fig. 30) but does not occur in simulations having a straight eastern plateau edge (e.g., Fig. 4). This indicates that a curved eastern edge geometry of the Caribbean plateau allow for separation of the Greater Antilles Arc from the Caribbean plateau by weakening the interface.

1.5 The role of a composite plateau

The main consequences of having a composite plateau are observed in the simulations including a weak zone separating the aggregated sub-plateaus i.e. simulations CP.1 to 4 and CP.7 to 8 (Figs 6, 7, 8,9,12 and 13).

For instance, simulation CP.2 (Fig. 7) shows that shortly after collision of the eastern edge of the plateau with the proto-Caribbean margin, separation of the southernmost sub-plateau triggers upwelling of the asthenophere and roll-back of the Farallon subduction along the northwestern South American margin (Fig. 7). At the same time, a segment of the Greater Antilles Arc is transported nearly 1000 km south along the South American margin. In this model, subduction is not yet resumed on the western edge of the plateau by the end of the simulation.

Simulation CP.1 (Fig. 6) uses a slightly stronger mantle rheology for the plateau aggregate (V = 14.00×10^{-6} m^3.mol^{-1} instead of V = 13.50×10^{-6} m^3.mol^{-1} for simulation CP.2). The slightly stronger plateau mantle helps to stabilize the system and, contrary to simulation CP.2, subduction renewal is

observed on the eastern edge of the disconnected southern sub-plateau (Fig. 6). Subsequently, subduction-driven plume triggers voluminous partial melting in front of the rolling-back proto-Caribbean slab in a similar manner to what is observed for the reference simulation (MP.1, Fig. 14). Note that for simulation CP.1, a section of the Greater Antilles Arc is also transported onto the rolling back proto-Caribbean plate.

In simulation CP.5, the sub-plateaus are separated by oceanic crust instead of weak zones. This results in a geodynamic evolution rather similar to the reference model (MP.1, Fig. 14) with the exception of having a more efficient transfer of the plateau along the northern margin of South America (Fig. 10). We note that such a geometry gives a better fit with the actual geometry of central American margin although, in this simulation, no fragment of the Greater Antilles Arc is transported onto the rolling back proto-Caribbean plate.

Simulation CP.7 (Fig. 12) investigate a similar sub-plateaus organization with respect to simulation CP.5. In this simulation, however, narrow weak zones separate the sub-plateaus with the larger weak zone placed between the two northern sub-plateaus. An interesting feature in this model can observed after collision of the plateau, during the initial stage of roll back of the proto-Caribbean plate: the plateau aggregate is breaking up and the easternmost sub-plateau is dragged with a fraction of the Greater Antilles Arc onto the rolling back proto-Caribbean plate. Subsequently, during subduction renewal on the western edge of plateau, the plume is triggered in the break-up area, with a plume head directed toward the subducting Farallon plate (Fig. 12D) instead of toward the proto-Caribbean plate as recorded for the reference model (MP.1, Fig. 14).

Overall, the simulations including a composite plateau shows that relatively small differences in the initial geometry/mantle rheology can largely control second order features such as the fragmentation of the plateau and the geometry of the plume during subduction renewal (e.g. simulation CP.7, Fig. 12), the geometry of the central America margin after subduction renewal (e.g. simulation CP.6, Fig. 11), and the volume of accreted material during plateau transfer on the northern South America margin (e.g. simulation CP.5, Fig. 10).

References

[1] Kerr, A.C.: Oceanic plateaus. Treatise on geochemistry **4**, 631–667 (2014)
