# Supplementary material for: Subduction initiation triggered the Caribbean large igneous province
Source: Nat Commun. 2023 Feb 11;14:786. doi: 10.1038/s41467-023-36419-x (PMC9922256; doi:10.1038/s41467-023-36419-x)
Supplement: Supplementary file 3 — Description to Additional Supplementary Information [file 41467_2023_36419_MOESM3_ESM.pdf]

Description of Additional Supplementary Files

Video: “Movie of the reference model showing the 3D geodynamic evolution of the Cretaceous Caribbean system”
